# The Arabic Version of the Patient Health Questionnaire-2 (PHQ-2): Psychometric Evaluation Among Mothers of Children With Intellectual Disabilities

**DOI:** 10.1155/nrp/9934710

**Published:** 2025-07-10

**Authors:** Amira Mohammed Ali, Saeed A. Al-Dossary, Musheer A. Aljaberi, Heba Emad El-Gazar, Carlos Laranjeira, Haitham Khatatbeh, Mohamed Ali Zoromba, Rasmieh Alamer, Faten Amer, Annamaria Pakai, Feten Fekih-Romdhane

**Affiliations:** ^1^Department of Psychiatric Nursing and Mental Health, Faculty of Nursing, Alexandria University, Smouha, Alexandria 21527, Egypt; ^2^Department of Psychology, College of Education, University of Ha'il, Ha'il 1818, Saudi Arabia; ^3^Research Centre Innovations in Care, Rotterdam University of Applied Sciences, Rotterdam, the Netherlands; ^4^Department of Internal Medicine, Section Nursing Science, Erasmus University Medical Center (Erasmus MC), Rotterdam 3015 GD, the Netherlands; ^5^Department of Nursing Administration, Faculty of Nursing, Port Said University, Port Said, Egypt; ^6^School of Health Sciences, Polytechnic University of Leiria, Campus 2, Morro do Lena, Alto do Vieiro, Apartado 4137, Leiria 2411-901, Portugal; ^7^Centre for Innovative Care and Health Technology (ciTechCare), Polytechnic University of Leiria, Campus 5, Rua das Olhalvas, Leiria 2414-016, Portugal; ^8^Comprehensive Health Research Centre (CHRC), University of Évora, Évora 7000-801, Portugal; ^9^Faculty of Nursing, Yarmouk University, Irbid, Jordan; ^10^College of Nursing, Prince Sattam bin Abdulaziz University, Al-Kharj, Saudi Arabia; ^11^Psychiatric and Mental Health Nursing Department, Mansoura University, Mansoura, Egypt; ^12^Faculty of Pharmacy, An-Najah National University, Nablus, State of Palestine; ^13^Institute of Nursing Sciences, Basic Health Sciences and Health Visiting, Faculty of Health Sciences, University of Pécs, Pécs 7622, Hungary; ^14^Faculty of Medicine of Tunis, Tunis Al Manar University, Tunis, Tunisia; ^15^Department of Psychiatry Ibn Omrane, The Tunisian Center of Early Intervention in Psychosis, Razi Hospital, Tunis, Tunisia

**Keywords:** Arabic/Saudi, cutoff score/cutoff point, depression/low mood, mothers/children with mental or intellectual disabilities, Patient Health Questionnaire 2-item scale/PHQ-2, psychometrics/construct validity, receiver-operating characteristic curve/ROC analysis, stress/sleep/nightmares/physical health/psychological support

## Abstract

**Aim:** Mothers of children with intellectual disabilities are particularly vulnerable to mental distress due to demanding and exhausting caregiving. However, in the Arab world, they are seldom screened for depression because of limited diagnostic resources. Addressing the urgent need for brief and reliable screening tools, this study evaluated the psychometric properties of the Arabic version of the Patient Health Questionnaire-2 (PHQ-2) among 85 Saudi mothers.

**Design:** A cross-sectional study.

**Methods:** The construct, convergent, and divergent validity of the PHQ-2 was examined through a latent variable model (LVM), while its cutoff score was examined through receiver-operating characteristic (ROC) curve analysis.

**Results:** The unidimensional PHQ-2 (item loadings > 0.7) was positively predicted by stress and negatively predicted by high mood and happiness, supporting its convergent and divergent validity. The PHQ-2 effectively predicted low mood, poor sleep quality, nightmares, high stress, low general physical health, and willingness to join a psychological support program (area under the curve [AUC] range = 0.72–0.84, *p* values < 0.001). The best balance between sensitivity and specificity was achieved at the PHQ-2 threshold ≥ 2.5, while the cutoff ≥ 3.5 demonstrated a higher positive predictive value (PPV) for all outcomes (range = 30.0–78.8 vs. 23.0–70.8).

**Conclusions:** The PHQ-2 is a brief, valid tool, which at cutoffs ≥ 2.5 and ≥ 3.5 can reliably detect clinically significant depression and related psychological and physical adverse effects. Mothers scoring ≥ 3.5 may require a clinician-based examination for depression, and they may benefit from specific mental health literacy interventions. However, the results should be interpreted with caution given convenience sampling, a small sample size, and elevated distress levels in the current population. These limitations highlight the need to replicate the study with larger, randomly selected samples from more diverse populations.

**Implications for Practice:** Nurses can efficiently screen for depression and its mental/physical sequelae, as well as monitor response to treatment using only two items. The study provided two well-interpreted cutoffs of the PHQ-2, with real-world implications for mental health screening in under-resourced settings.

**Reporting Method:** The study adhered to STROBE guidelines.

**Patient or Public Contribution:** No patient or public contribution.

**Summary**

• What This Paper Adds:
∘ Validating a brief scale for use in busy and resource-limited clinical settings.
∘ The PHQ-2 may lower nonresponse and delayed diagnosis of depression in 22 Arab countries.

## 1. Introduction

Depression is a multifactorial disorder that arises from a complex interplay among genetic, sociodemographic, lifestyle, personality, and environmental factors [1, 2]. Major depressive disorder (MDD), one of the top 10 causes of years lost to disability and a second cause of global disease burden, prevails in 5% of the general population—up to 300 million people worldwide [2–5]. Its prevalence has exponentially increased over the last few decades (by up to 150%) [2, 5]. Depression entails a condition of generalized inflammation and oxidative stress, which promotes the development and exacerbation of numerous physical and mental illnesses such as coronary heart disease, diabetes, and Alzheimer's disease [4, 6]. Depression is frequently undetected/untreated, especially in low- and middle-income countries [3, 7]. This is probably due to lack of mental health literacy (poor knowledge of a mental disorder and stigma), which discourages early detection of symptoms and engagement in culturally effective education and treatment [8]. Validated brief measures that reliably identify depression are essential to mitigate its adverse effects on health, quality of life, and productivity [5, 8].

Caring for children, especially those with intellectual disabilities, can be highly stressful [9]. Those children frequently exhibit poor sleep symptoms as well as disruptive behaviors (irritability and running out of the house at night) [10]. Therefore, the mothers are continually alert, and most of them sleep with their children in the same room or the same bed. Accordingly, they may spend a substantial proportion of nighttime in caregiving [11]. Mothers may also be burdened with working outdoors to support their living, in addition to the demands of caring for their healthy children and meeting their social and family responsibilities, which put their psychological and physical wellbeing at jeopardy [12].

Sleep problems such as insomnia, difficulty initiating/maintaining sleep, are highly prevalent worldwide, affecting up to 22% of adults. Daytime impairment is reported in approximately 15% of those affected [13]. Nightmares are distressing mental imagery, which evoke nocturnal awakening, nonrestorative sleep, and sleep avoidance. They occur weekly in 2%–5% of adults, and they are highly associated with other sleep disturbances such as insomnia [12, 13] and poor sleep quality—low levels of self-perceived satisfaction with sleep. Caregiving women are at a great risk of poor sleep and nightmares secondary to their children's poor sleep, disruptive behaviors, and caregiving stress, particularly at nighttime [11, 12]. Longitudinal and experimental studies show that chronically poor sleep increases the risk of developing future depression [2, 14, 15]. It operates through a mechanism that impedes the removal of brain wastes, giving rise to neuronal damage [15]. Additionally, poor sleep is associated with adverse psychological effects such as cognitive decline and poor emotional regulation, which promote the development of depressive symptoms over time [16].

Poor sleep also increases vulnerability to poor physical health, which manifests by uncomfortable symptoms (e.g., fatigue, headache, gastrointestinal discomfort) and physical disorders (e.g., diabetes, hypertension) [11]. In line, poor physical health predisposes to defective coping and mental frailty [17]. On the other hand, depression promotes the development and deterioration of up to 29 physical morbidities (e.g., of the endocrine, musculoskeletal, and circulatory systems) by discouraging self-care, help-seeking, and adherence to prescribed treatments [4–18]. Indeed, mothers of children with intellectual disabilities and their children experience frequent hospitalization. During their children's admission (e.g., to critical care units), the mothers remain actively involved in caregiving, often with limited perceived support from healthcare professionals, which negatively impacts the continuity and quality of their sleep [19].

Among various common measures, the Patient Health Questionnaire 9-Item scale (PHQ-9) tightly matches the DSM-IV diagnostic criteria for depression. Despite its relative brevity, it may not yield the required response when used frequently in some clinical settings because of time constraints [20]. Research indicates that two items from the Primary Care Evaluation of Mental Disorders (PRIME-MD) Screening Questionnaire, which address depressed mood and loss of interest, are used in general practice, and they may effectively capture depression. However, the binary nature (yes/no) of these items may underestimate the severity of depression or the extent of symptom change over time. Thanks to its four-point response format, the PHQ-2 may overcome these shortcomings [21]. Many studies testing the PHQ-2 diagnosed MDD through standard diagnostic methods such as the Composite International Diagnostic Interview (CIDI) [3], the Mini International Neuropsychiatric Interview (MINI) [20], and the Structured Clinical Interview for DSM-IV (SCID) [21, 22]. The performance of the PHQ-2 was also favorable relative to the PHQ-9 and other long measures of depression such as Beck Depression Inventory [20–23]. It successfully reflected response to treatment [20] as well as longitudinal changes in the course of depression, e.g., spontaneous remission, resistance/lack of change, or condition deterioration [21]. However, reports on its cutoff scores, which range from ≥ 2 to ≥ 3, are inconsistent in terms of the accuracy of their diagnostic performance [23, 24].

The PHQ-9 and its short form PHQ-2 have been validated in up to 80 languages [3]. Until the current moment, psychometric testing is entirely lacking for the PHQ-2 in Arabic—a language spoken by around 500 million people in 22 countries [25, 26]. Moreover, economic and political instabilities in the Middle East and North Africa triggered the displacement of large numbers of Arabs into the USA, Australia, France, Germany, the UK, among others [27, 28]. Mental and physical health problems are documented among immigrants and refugees, stressing the need for well-tested Arabic measures [29]. Therefore, this study addresses a previously unexplored context by evaluating the psychometric properties of the Arabic version of the PHQ-2 as a brief measure for diagnosing depression. As per the literature, it hypothesized that the Arabic PHQ-2 would have a unidimensional structure, which would strongly correlate with measures of mood, stress, poor sleep, nightmares, and poor general physical health. The study included a measure of happiness to test the divergent validity of the PHQ-2. It also aimed to test its criterion and predictive validity by determining its cutoff in relevance to the aforementioned constructs.

## 2. Methods

### 2.1. Design and Participants

Using an anonymous online survey, this cross-sectional study recruited a convenience sample of 85 Saudi mothers of children with intellectual disabilities. Invitations to take part in the study were disseminated through parent groups on Facebook and WhatsApp as well as advertisements posted in special childcare centers in the Kingdom of Saudi Arabia. Potential participants were informed about the commencement of a free psychological support intervention. Only mothers were encouraged to take part. Participants eligible for inclusion in the study were those aged 18 years or older who had children with intellectual disabilities, could speak Arabic, and owned a smartphone. They were asked to complete the survey even if they did not wish to take part in the supportive intervention. Before completing the survey, they signed a digital informed consent form, which reassured them about voluntary participation and confidentiality of their data. The study protocol was approved by the Research Ethics Committee of Ha'il University (11/9/2023: H-2023-367).

### 2.2. Measures

A self-administered questionnaire was used for data collection. It elicited information on the sociodemographic characteristics of the mothers (age, marital status, desire to take part in a support program). The questionnaire had many sections, which explored the clinical characteristics of the participants including the following measures:

The Patient Health Questionnaire-2 (PHQ-2) is a two-item version of the PHQ-9. Its items measure depressive symptoms over the last two weeks: “losing interest” and “feeling down, depressed, or hopeless.” Items are rated on a four-point response format ranging from 0 (*not at all*) to 3 (*almost every day*). The minimum and maximum total scale scores range from 0 to 6; higher scores reflect greater symptom severity [21].

Mood was assessed by a single item “Rate your mood on a scale from 0 (*very bad/the worst mood ever*) to 10 (*excellent/the best mood ever*).” Higher scores of this item reflect good mood and vice versa [30].

Happiness was assessed by a single item “Rate the extent to which you generally feel happy over the past week on a scale from 0 (*very unhappy*) to 10 (*extremely*).” Higher scores reflect greater happiness [31].

Stress was assessed by a single item “In the past year, how would you rate the amount of stress in your life (at home and at work/study)?” Mothers were requested to rate their experience on a scale ranging from 1 (*no stress*) to 6 (*extreme stress*) [32].

Sleep quality was assessed by a single-item measure: “Rate the quality of your sleep on a scale from 0 (*very poor*) to 10 (*excellent*)” [33].

Perceived physical health was assessed by a single item “How do describe your general physical health?” They rated their response on a five-point scale ranging from 1 (*poor*) to 5 (*excellent*) [34].

Mothers were asked to describe their experience of dreams during the past month by a single item (no dreams/neutral content and bad dreams/nightmares) [12]. Mothers' desire to participate in a supportive program was assessed by a single binary (yes, no) item.

### 2.3. Statistical Analysis

Frequencies and percentages were used to describe categorical variables. The distribution of quantitative variables was checked. Accordingly, normally and nonnormally distributed variables were reported as mean ± standard deviation and median (interquartile range: Q1–Q3), respectively.

The internal consistency of the PHQ-2 was measured by coefficient *α*, while item–total correlations were used to address its convergent validity at the item level. We calculated intraclass correlation coefficients (ICCs) with 95% confident intervals using mean-rating (*k* = 85), absolute-agreement, and two-way random-effects model [35, 36].

Confirmatory factor analysis (CFA) of scales that comprise fewer than four items results in noninformative saturated models, i.e., the values of chi-square (*χ*^2^) and degree of freedom (DF) equal zero [7, 37]. As a remedy, the CFA model capturing the construct validity of the PHQ-2 was nested within a latent variable model (LVM) by anchoring it to the measures of mood, stress, and happiness, which were included as exogenous variables in the model. This approach also allows testing the convergent and divergent validity of the PHQ-2 [27]. Considering the model as well as fitting was based on the thresholds of < 3, < 0.05, and > 0.95 for the following fit indices: *χ*^2^/DF (CMIN/DF), (root-mean-square error of approximation [RMSEA] and standardized root-mean-square residual [SRMR]),and (Comparative Fit Index [CFI] and Tucker–Lewis Index [TLI]), respectively [1, 25].

For predictive validity testing, receiver-operating characteristic (ROC) curve analysis was conducted using the PHQ-2 as a continuous variable to predict low mood, poor sleep quality, nightmares, poor physical health, stress, and willingness to join a supportive intervention. For this purpose, the categorical form of the continuous outcome variables was obtained by categorizing them according to their quartile scores (Table 1): low mood and poor sleep quality < 4, poor physical health < 3, and high stress ≥ 5—this is because quartile scores may operate to a great extent like statistically determined cutoff scores [1, 37].

The diagnostic potential of the PHQ-2 was considered based on various fit indices of different ROC models: area under the curve (AUC), sensitivity and specificity for all possible cutoff points, and the Youden index (sensitivity + specificity) − 1 [1, 18]. In the next step, two categorical forms of the PHQ-2 were defined based on cutoff points resulting from the analysis (≥ 2.5 and ≥ 3.5). Then, *χ*^2^ index was used to determine the positive predictive values (PPVs) and the negative predictive values (NPVs) of each threshold. The analyses were performed in SPSS Version 22 and Amos Version 26. Significance of the results was set at probability levels below 0.05 in two-tailed tests.

## 3. Results

Our mothers had a mean age of 40.8 ± 8.3 years. Most of the respondents were married (87.1%) and willing to receive psychological support (63.5%). More than one-fifth of the mothers (22.4%) frequently experienced nightmares. Table 1 displays the clinical characteristics of the mothers.

As shown in Table 2, the frequency of losing interest (Item 1) was greater than that of feeling blue, depressed, or hopeless (Item 2). Both items had strong significant positive correlations with each other and with the total score of the PHQ-2, indicating scale homogeneity and convergent validity at the item level.

**Figure 1:**
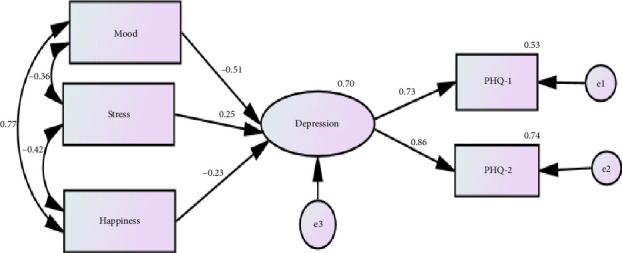
Latent variable model exploring the construct (convergent and divergent) validity of the Patient Health Questionnaire-2 (PHQ-2) among mothers of children with intellectual disabilities.

The LVM displayed excellent fit (*χ*^2^ = 3.01, DF = 3, *p* = 0.390, CMIN/DF = 1.0, CFI = 0.999, TLI = 0.999, RMSEA = 0.007, RMSEA 95% CI: 0.000 to 0.184, SRMR = 0.025). It predicted 70% of the variance in depression.  Figure 1 shows that the loadings of both items of the PHQ-2 were high, indicating strong correlation with their domain-specific factor, i.e., adequate construct validity. Convergent validity was expressed by a significant direct positive effect of stress on the PHQ-2. Because higher scores of the single item measuring mood reflect good mood, this item had a significant negative direct effect on the PHQ-2 (i.e., higher PHQ-2 scores reflect low mood), lending further support to its concurrent/convergent validity. Meanwhile, divergent validity was expressed by a significant direct negative effect of happiness on the PHQ-2.

The reliability of the PHQ-2 was adequate (coefficient alpha = 0.76). The values of item–total correlations were also high (*r* = 0.61), indicating adequate convergent validity at the item level. The coefficient alpha of the PHQ-2 was comparable to the average measures ICC, indicating good scale reliability. In contrast, the single measures ICC was at the lower end of the acceptable range (0.60, 95% CI: 0.45–0.73), suggesting potential variability due to rater error.

**Figure 2:**
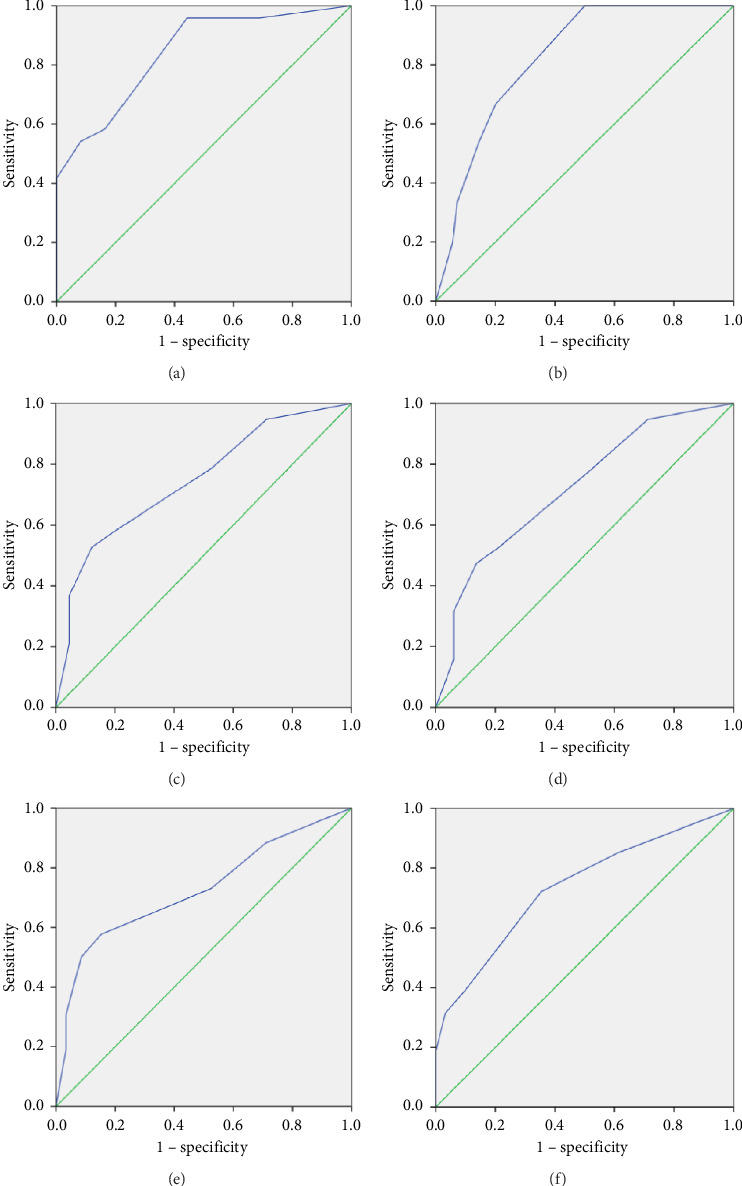
Receiver-operating characteristic (ROC) curve exploring the predictive/criterion validity of the Patient Health Questionnaire-2 (PHQ-2) among mothers of children with intellectual disabilities. (a) Mood. (b) Sleep quality. (c) Nightmares. (d) General physical health. (e) Stress. (f) Joining a supportive program.

The results of predictive/criterion validity as tested by the ROC analysis are shown in Table 3 and Figure 2. For all outcomes, the best balance between the highest values of both sensitivity and specificity was achieved at a PHQ-2 threshold of ≥ 2.5. AUC values indicated good diagnostic accuracy of the PHQ-2 for mood and sleep quality. The diagnostic accuracy of the PHQ-2 was moderate for nightmares, general physical health, stress, and joining a supportive program. Sensitivity values were high for all outcomes, albeit specificity values were lower, particularly for joining a supportive program.

## 4. Discussion

Brief measures enable the efficient monitoring of psychiatric symptoms, which may be routinely performed to identify unmet treatment needs. This practice would enhance the quality and effectiveness of psychological interventions while offering essential evidence of service efficacy to insurance providers and funding agencies [20]. Research emphasizes that the performance of the PHQ-2 is favorable relative to standard diagnostic interviews and well-established measures of depression; it is also sensitive to change (e.g., in response to treatment or spontaneous remission) [3, 20–24, 38]. Filling a gap in the current literature, this study is the first to examine the psychometric properties of the PHQ-2 in an Arab culture and among mothers of children with disabilities. Our LVM confirms the unidimensional structure of the scale as well as its convergent and divergent validities. Examining its cutoff relative to six outcome criteria shows that the PHQ-2 at the cutoff point ≥ 2.5 achieved the best balance between the highest values of both sensitivity and specificity, albeit higher PPV was noticed at the cutoff ≥ 3.5 (Table 3).

Different values of the optimal cutoff score of the PHQ-2 have been reported in the literature: ≥ 1 among Spanish Speaking Immigrants in Chile [3], ≥ 2 among Spanish primary care patients and Iranian students [5, 22]; and ≥ 3 among Italian cardiovascular inpatients, German medical outpatients, and Australian recipients of internet-delivered cognitive behavior therapy for common mental disorders [20, 21, 39]. Such variations are congruent with notions that cutoff scores are not universal, and they should be determined for each region and for each disease condition [40]. In our sample, the PHQ-2 mean score (Table 1) was greater than previously recorded cutoffs for detecting clinically significant depression in people with physical and mental conditions, i.e., ≥ 3 cardiovascular disorders and common mental disorders [20, 21, 39]. Meanwhile, classifying the respondents based on the cutoffs detected in the present study indicates that 76.5% and 58.8% of our mothers had PHQ-2 scores ≥ 2.5 and ≥ 3.5, respectively. Those scoring above the cutoffs are typically referred to the advanced diagnostics of depression. However, a cutoff of 2.5 may be less favorable in under-resourced settings due to the high cost of advanced diagnostic procedures. Nevertheless, it is crucial not to overlook any case of depression during screening, and a lower PHQ-2 cutoff (≥ 2) demonstrated effectiveness in achieving this goal [23, 24].

For all outcomes, the PHQ-2 at the threshold ≥ 3.5 still had acceptable sensitivity (Table 3). Particularly, sensitivity remained the same as that noticed at the threshold ≥ 2.5 for two cordial outcomes: low mood and poor sleep quality. However, at this threshold, specificity considerably decreased for the rest of the outcomes, ranging from 0.44 to 0.53. This was subsequently followed by reductions in the Youden index. According to the criteria of cutoff selection, which count on maximizing both sensitivity and specificity, this denotes reduction in the optimal diagnostic performance of the scale at a cutoff of 3.5. This result is consistent with that of a meta-analysis, which shows that 19 out of 21 studies report a PHQ-2 cutoff ≥ 3 with pooled sensitivity and specificity of 0.76 (95% CI = 0.68–0.82) and 0.87 (95% CI = 0.82–0.90), respectively [24]. However, it is not possible to concomitantly increase both sensitivity and specificity for increasing cutoff scores of a given test that has continuous (or multiple) results. A test that is more specific, i.e., with increased cutoff value and diagnostic potential for true positive cases (PPV), will be a less sensitive test, i.e., with a reduced capacity for identifying true negative cases [40]. In this respect, sensitivity and specificity of the PHQ-2 cutoff ≥ 2 pooled from 17 studies (0.91 (95% CI = 0.85–0.94) and 0.70 (95% CI = 0.64–0.76), respectively) were associated with considerably lower heterogeneity than the cutoff ≥ 3 (*I*^2^ = 43.2% vs. 81.8%) [24]. However, in a previous study, PHQ-2 cutoffs that achieved the best balance between sensitivity (73%) and specificity (89%) at an AUC signifying excellent fit (0.85) exhibited exceptionally poor PPV (4%) despite the optimal NPV (100%) [3]. PPV is generally the metric of most utility in applied contexts, and low PPV indicates that the scale detects too many false positives [41]. Low mood is a main depressive symptom, and poor sleep is also a symptom that commonly manifests in depression and closely related sleep disorders [16, 21]. Thus, based on the greater benefit of the test “ruling-out the disease,” other indicators were considered in our trade-off for the higher cutoff: PPV and NPV were higher at the cutoff ≥ 3.5 for outcomes most relevant to MDD (low mood and poor sleep quality)—sensitivity remained high (Table 3), ensuring that most cases of depression were accurately identified. High PPV and NPV signify that the scale does not detect too many false positive or false negative cases [42]. The PHQ-2 cutoff score of ≥ 3.5 demonstrated greater specificity for these two outcome variables, increasing the likelihood of correctly identifying true cases of depression while minimizing false positives. This may reduce reliance on more costly confirmatory diagnostic procedures. Thus, this cutoff would be more convenient in our Arab context, which is low in investments focused on diagnosing and treating mental diseases. In other words, it may not be possible/acceptable in low resource settings to consider lower cutoffs that address cases with slight severity [7, 43]. Additionally, research associates intense depressive symptoms with low sleep quality, which frequently follows nightmares and ruminations over caregiving-related stressors [12]. Congruent with the literature, PPV of the PHQ-2 at the cutoff ≥ 3.5 for all the outcomes was higher than that of the cutoff ≥ 2.5, with slight reductions in NPV (Table 3), suggesting that this score is still capable of identifying those who frequently experience nightmares, poor physical health, or high stress.

The PHQ-2 mean score was also higher among those who intended to participate in the psychological support program (Mann–Whitney *U* = 442.5, *z* = −3.69, *p* < 0.001). However, mothers' willingness to take part in the support program was slightly higher among those who scored ≥ 2.5 than those who scored ≥ 3.5 on the PHQ-2 (54.1% vs. 45.9%). In line, those with higher scores of depression/anxiety were less likely to seek help (e.g., for pain treatment) because they may consider treatment as ineffective [18]. Severe depression is associated with greater alterations in mental processes involving poor self-efficacy (i.e., that they can seek help), focusing on negative events in past treatment experiences (e.g., drug resistance), lack of expression of negative emotions, lack of awareness of suicidal behaviors, and possibly greater stigma [44–46]. Therefore, those scoring ≥ 3.5 on the PHQ-2 may benefit from educational interventions that focus on depression literacy and help seeking [45].

### 4.1. Strength, Implications, and Limitations

This study, which adheres to STROBE reporting guidelines, has the merit of addressing a previously unexplored research area: the psychometric performance of PHQ-2 in Arabic and in a highly distressed sample—caregiving mothers of children with intellectual disabilities. It employed methodologically robust statistical approaches (LVM and ROC analysis) for evaluating the construct, convergent, and divergent validities of the scale and determining its cutoff. The results confirm the usability of the PHQ-2 for predicting low mood as well as distressful psychological problems such as poor sleep quality, nightmares, stress, and poor physical health. Accordingly, mothers scoring ≥ 2.5 should undergo advanced assessment for mental morbidities, while those scoring ≥ 3.5 should be given a higher priority in resource-limited settings. Because the PHQ-2 is ultra-brief, it may be combined with other brief measures to evaluate a wide range of outcomes that may be crucial for the assessment of depression and its management in routine care and research.

The study comes with some limitations, which should be considered while interpreting the results as they may limit their generalizability. While former studies [3, 20–23] compared the properties of the PHQ-2 relative to the full-length scale, other lengthy measures of depression, or diagnostic interviews (e.g., CIDI, MINI, SCID), a major limitation in our study is that no gold-standard diagnostic tool was used to confirm depression diagnosis—because of access and logistic constraints, it was not possible to have a clinician-based diagnosis for concurrent validity testing, especially as the mothers did not attend mental or public health settings. Alternatively, we counted on a single-item measure of mood. Brief measures of all other outcome measures were used to overcome poor response rates, which are evident in surveys in the Arab region [28]—particularly, mothers are busy spending a long time in childcare and may be reluctant to contribute because they do not confide in surveys or perceive them as personally useful [47]. It is true that single-item measures cannot undergo reliability or construct validity testing, but these measures may capture global or unidimensional constructs at predictive validity equal to specific multiple-item measures [48, 49]. In agreement with this logic, cutoffs revealed by our analysis are comparable to those previously reported in studies validating the PHQ-2 against standardized clinical interviews [23, 24], which casts credibility on cutoffs estimated in the current analysis. Nevertheless, it is necessary that future studies overcome this major limitation by clinically validating the diagnosis of depression.

The study is also amenable to the risk of selection bias for many reasons. The sample size was relatively small (*N* = 85). However, according to the role of thumb, which requires 10 responses per item for testing the structure of a tool [50], this sample may credibly support the testing of our model, which comprised eight items in total. On the other hand, the characteristics of the participants may diverge from the general mother population as we included only those who can use smart phones to access the internet (educational level, seeking information, and support for self-care/childcare). Despite disseminating the link of the survey through Facebook groups of the parents of special need children in other countries (Jordan, Egypt, Kuwait, Qatar, Morocco, and Yemen), we failed to obtain responses from any of those countries. Owing to the help of childcare centers that were personally contacted by the second author, the sample came only from Saudi Arabia. Saudi Arabia is a large country with a great heterogeneity in socioeconomic standards, urbanization, educational (e.g., special childcare) and healthcare services, customs, support systems, among others [51]. Therefore, our nonrandom sample may not even represent Saudi and Arab mothers of children with intellectual disabilities. Future studies that overcome these limitations will provide credible information on the psychometrics of the PHQ-2.

## 5. Conclusions

The Arabic PHQ-2 is a reliable unidimensional measure, which demonstrates adequate convergent and divergent validities. The best balance between sensitivity and specificity in the ROC analysis revealed two thresholds of the PHQ-2 (≥ 2.5 and ≥ 3.5). The latter demonstrated considerable reductions in specificity for different outcomes except for two outcomes that are tightly related to MDD (low mood and poor sleep quality). Nonetheless, it had slightly higher PPV for all outcomes than the cutoff ≥ 2.5. Accordingly, the cutoff ≥ 2.5 may be used in depression screening to avoid missed diagnosis, while PPV denotes that the cutoff ≥ 3.5 may be more accurate for diagnosing severe form/clinically significant depression as it manifests primarily with low mood as well as poor sleep quality. Further investigations of the PHQ-2 in relevance to lengthy measures of depression or standardized diagnostic interviews in larger samples from other Arab countries may support these conclusions.

## Figures and Tables

**Table 1:** Clinical characteristics of the participants (*N* = 85).

Characteristics	Mean ± SD	MD (Q1–Q3)
PHQ-2	3.2 (1.1)	4 (3–5)
Mood	5.7 (2.9)	6 (4–8)
Sleep quality	5.6 (2.5)	5 (4–7)
Perceived physical health	4.0 (1.8)	3 (3–4)
Stress	3.5 (1.7)	3 (2–5)
Happiness	6.0 (2.9)	6 (5–8)

*Note:* MD: median and PHQ-2: Patient Health Questionnaire 2-item scale.

Abbreviation: SD, standard deviation.

**Table 2:** Item analysis and descriptive statistics of the Patient Health Questionnaire-2 (PHQ-2) among mothers of children with intellectual disabilities (*N* = 85).

Items	Mean (SD)	Median (Q1–Q3)	Skewness (SE)	Kurtosis (SE)	Shapiro–Wilk	Correlations	
						1	2
1. Little interest or pleasure in doing things	2.08 (1.04)	2 (1–3)	0.62 (0.26)	−0.76 (0.52)	0.83^∗∗^	—	
2. Feeling down, depressed, or hopeless	1.96 (0.97)	2 (1–2)	0.80 (0.26)	−0.29 (0.52)	0.82^∗∗^	0.607^∗∗^	—
3. Patient Health Questionnaire-2	4.05 (1.80)	4 (3–5)	0.81 (0.26)	−0.15 (0.52)	0.88^∗∗^	0.904^∗∗^	0.889^∗∗^

^∗∗^Correlations are significant at the level of 0.01.

**Table 3:** Cutoff scores of the Patient Health Questionnaire-2 (PHQ-2), along with goodness-of-fit indices associated with receiver-operating characteristic (ROC) curve analysis among mothers of children with intellectual disabilities (*N* = 85).

	AUC	SE	*p*	AUC 95% CI	Cutoff	Sensitivity	Specificity	Youden index	PPV%	NPV%	Cutoff	Sensitivity	Specificity	Youden index	PPV%	NPV%
Mood	0.84	0.05	0.001	0.75–0.94	2.5	0.96	0.69	0.65	35.4	95	3.5	0.96	0.44	0.40	46.0	97.1
Sleep quality	0.83	0.05	0.001	0.73–0.92	2.5	1.00	0.71	0.71	23.1	100	3.5	1.00	0.50	0.50	30.0	100
Nightmares	0.75	0.07	0.001	0.62–0.88	2.5	0.95	0.71	0.66	27.7	95.0	3.5	0.79	0.53	0.32	30.0	88.6
General physical health	0.72	0.07	0.004	0.59–0.85	2.5	0.95	0.71	0.66	27.7	95.0	3.5	0.79	0.53	0.32	30.0	88.6
Stress	0.73	0.07	0.001	0.60–0.85	2.5	0.89	0.71	0.60	35.4	85.0	3.5	0.73	0.53	0.26	38.0	80
Joining support program	0.74	0.05	0.001	0.63–0.84	2.5	0.85	0.61	0.46	70.8	60.0	3.5	0.72	0.36	0.08	78.8	57.1

Abbreviations: AUC, area under the curve; CI, confidence interval; NPVs, negative predictive values; PPVs, positive predictive values; SE, standard error.

## Data Availability

The data that support the findings of this study are available on request from the corresponding author. The data are not publicly available due to privacy or ethical restrictions.
